# EBV-associated lymphoepithelioma-like thyroid carcinoma with favorable outcome: case report with cytopathologic and histopathologic study

**DOI:** 10.1186/s13000-018-0713-0

**Published:** 2018-06-09

**Authors:** Chih-Yi Liu, Shih-Hung Huang

**Affiliations:** 1 Division of Pathology, Sijhih Cathay General Hospital, No.2, Lane 59, Jiancheng Road, Sijhih District, New Taipei City, 22174 Taiwan; 2 Department of Pathology, Cathay General Hospital, Taipei, Taiwan; 3 College of Medicine, Fu Jen Catholic University, New Taipei City, Taiwan

**Keywords:** Thyroid, Cytology, Lymphoepithelioma-like carcinoma, Epstein-Barr virus, EBER ISH

## Abstract

**Background:**

Lymphoepithelioma-like carcinoma (LELC) is a rare entity among thyroid tumors. Based on the limited number of case reports that exist, the association of Epstein–Barr virus (EBV) with primary thyroid LELCs seems inconsistent.

**Case presentation:**

We present a confusing cytological case of lymphoepithelioma-like thyroid carcinoma with expression of EBV. The patient presented with a central neck mass and bilateral lymphadenopathy. Fine-needle aspiration cytology revealed three-dimensional and syncytial fragments of epithelioid cells accompanied by small lymphocytes. The surgical specimen of resected thyroid tumor disclosed typical histopathological features of LELC. Metastatic papillary carcinoma was also discovered in the metastatic lymph nodes. In situ hybridization for EBV-encoded RNA (EBER-ISH) was positive in the tumor cells. Negative immunoreactivity for TTF-1, Pax-8, and CD5 was observed. The patient is currently undergoing regular follow-up and is 1 year and 10 months postresection with no evidence of recurrence.

**Conclusions:**

Long-term survival is discussed in relation to this variant of thyroid carcinoma, which might differ in behavior from anaplastic carcinoma. Further investigation is required to elucidate the clinical significance of EBV expression and progression of this unique variant of thyroid carcinoma.

## Background

Lymphoepithelioma-like carcinoma (LELC) is a subtype of thyroid carcinoma characterized by histologic features similar to those of undifferentiated carcinoma of the nasopharynx and LELC of other sites. Although LELC has been observed in many organs, primary LELC of the thyroid gland is extremely rare; only sporadic cases have been reported with emphasis on histopathological characteristics [1, 2]. This paper presents fine-needle aspiration cytology (FNAC) findings alongside histologic features in a patient with locally advanced and metastatic disease. Moreover, the patient exhibited a more positive response to radiotherapy with respect to long-term survival than is typical with conventional anaplastic thyroid carcinoma. The challenges associated with the cytological and histopathological findings are discussed. Additionally, we review this unique Epstein–Barr virus (EBV)-related malignancy and its possible impact on clinical outcomes.

## Case presentation

**Fig. 1:**
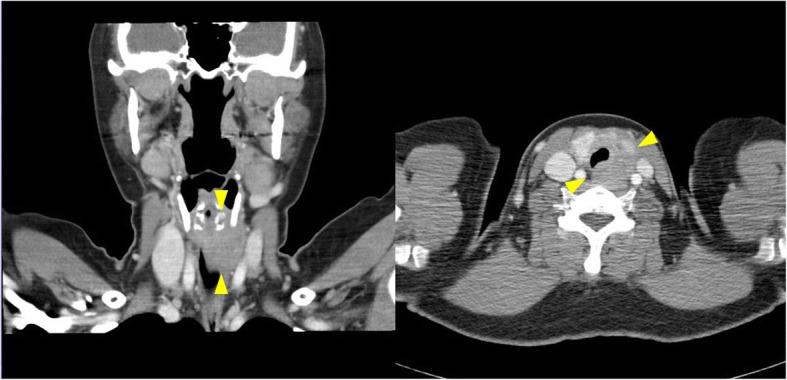
CT scan revealed a 4 cm hypodense mass with ill-defined margin at posterior-lower part of left thyroid gland (arrow heads), accompanied by multiple enlarged neck lymph nodes

**Fig. 2:**
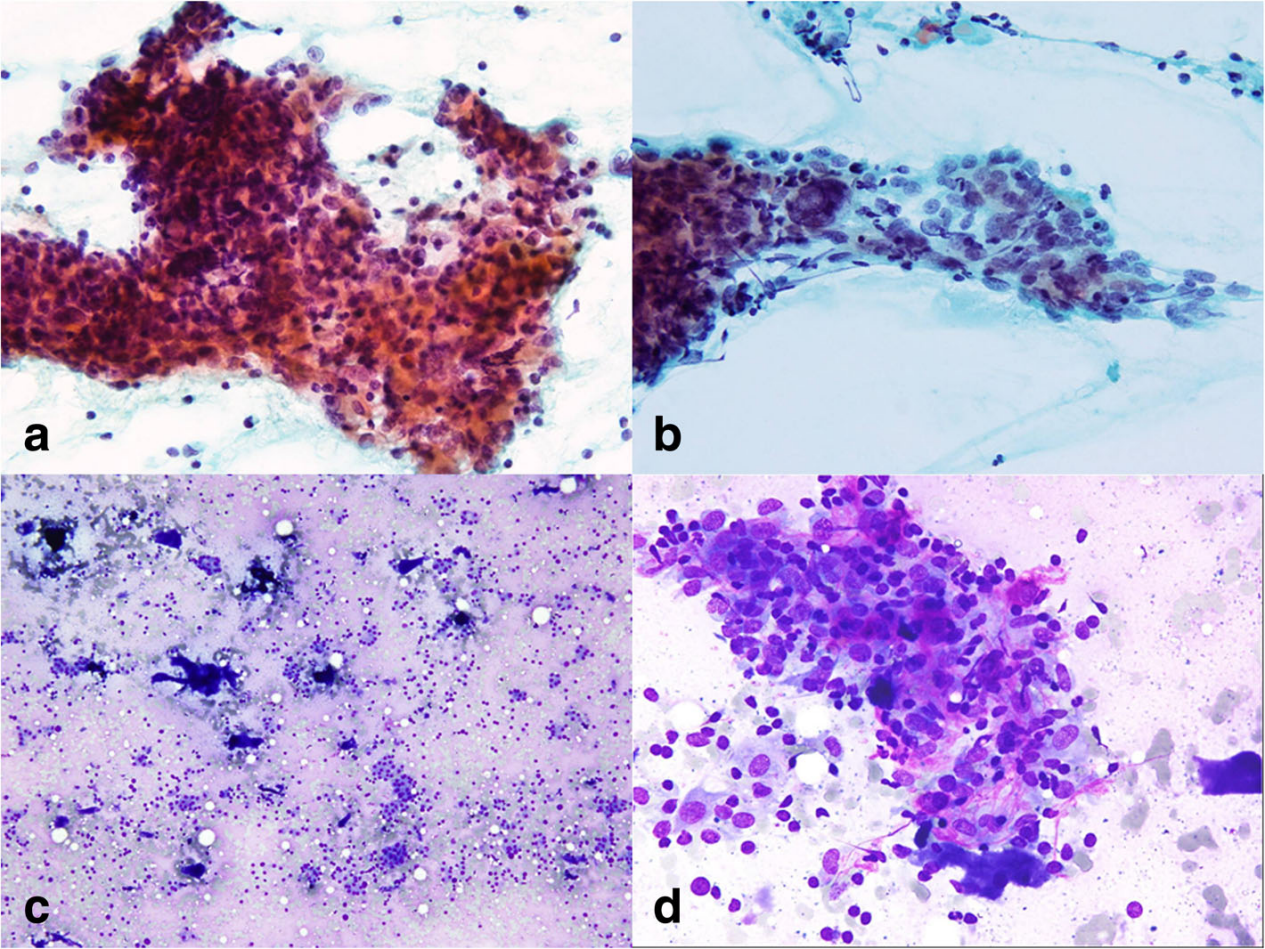
FNAC of neck lymph node: three-dimensional and syncytial cellular fragments accompanied by small lymphocytes (**a**. Papanicolaou, 400×); epithelioid cells with enlarged nuclei, vesicular chromatin, prominent nucleoli, and pleomorphic contours (**b**. Papanicolaou, 400×). FNAC of the left thyroid: cohesive clusters of follicular epithelial cells and dense extracellular matrix. (**c**. Liu, 100×); Few atypical epithelioid cells forming syncytial groups (**d**. Liu, 400×)

A 44-year-old female patient with a history of nodular goiter presented with progressive dyspnea and noisy breathing for several months. In ENT outpatient department, subglottic stenosis was discovered through endoscopy. A computed tomography (CT) scan demonstrated a 4.0 × 3.0 × 1.7 cm hypodense mass with ill-defined margins in the left thyroid gland (Fig. 1). The thyroid tumor was accompanied by three enlarged neck lymph nodes in the left level II and level III regions. Neither a nasopharyngeal lesion nor a lung mass was indicated through serial CT scans. The patient underwent FNAC for evaluation of the left neck lymph node and thyroid tumor. FNAC of the lymph node indicated cohesive epithelial fragments with syncytial appearance and lymphocytic infiltrates, resembling metastatic nasopharyngeal carcinoma (Fig. 2a and b). The FNAC sample of the left thyroid disclosed loosely cohesive clusters of follicular epithelial cells, as well as an abundant dense extracellular matrix (Fig. 2c). Only a few atypical epithelioid cells were recognized through the thyroid FNAC (Fig. 2d). The unusual presentation and cytomorphological features posed a diagnostic difficulty.

**Fig. 3:**
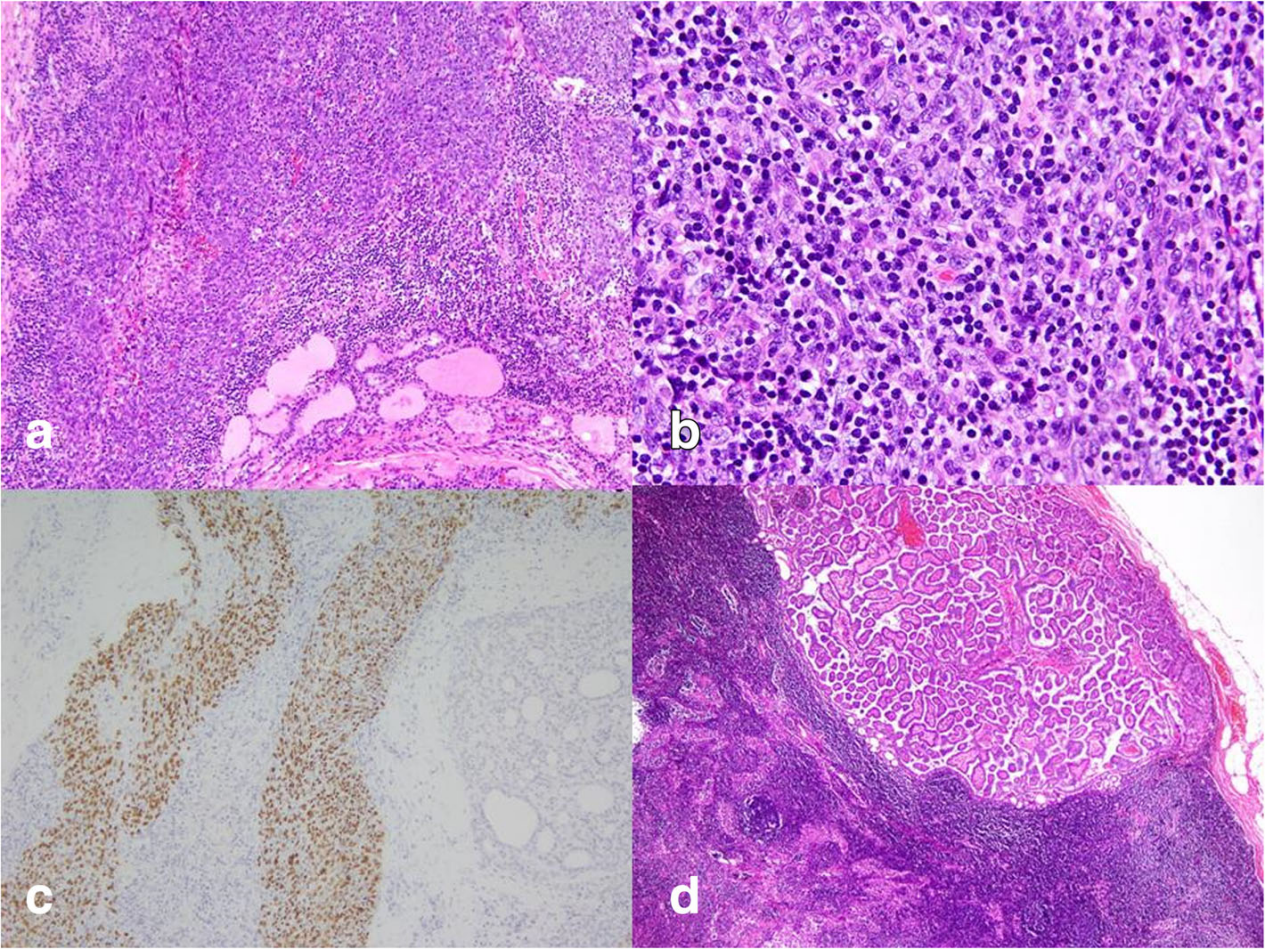
Histopathological findings of left thyroid tumor: sheets or nests of epithelial cells infiltrated by lymphoplasmacytic cells (**a**. H&E, 100X; **b**. H&E, 200×); malignant epithelial cells positive for EBER ISH (**c**. Hematoxylin counterstain, 200×); metastatic papillary carcinoma found in right neck lymph node (**d**. H&E, 40×)

Under the impression that a malignant thyroid tumor was present, the patient received debulking surgery, including a total thyroidectomy and bilateral neck lymph node dissection. The histologic slides of the left thyroid tumor revealed undifferentiated carcinoma with lymphoepithelioma-like features, (Fig. 3a and b) and a hyaline matrix was observed focally. The left and right thyroids were extensively sampled for characteristic components of well-differentiated carcinoma. Immunohistochemically, the tumor cells were positive for cytokeratin (AE1/AE3) and p40 but negative for TTF-1, Pax-8, and CD5. In situ hybridization for EBV-encoded RNA (EBER-ISH) was diffusely positive in the tumor cells (Fig. 3c). Seventeen and five lymph nodes were dissected from left and right soft neck tissue, respectively. The left metastatic node indicated metastatic carcinoma with lymphoepithelioma-like features, whereas metastatic papillary carcinoma was recognized in all of right neck lymph nodes (Fig. 3d).

The tumor could not be completely removed because of laryngeal invasion. Therefore, the surgeon performed a total thyroidectomy with preservation of the hypopharynx and larynx. Postoperative radiotherapy was provided for the gross residual local disease. After completion of adjuvant radiotherapy, a follow-up CT scan indicated a prominent regressive change in the tumor. At the time of study, the patient had been living with the disease for 1 year and 10 months.

## Discussion

LELCs exhibit a distinctive syncytial growth pattern with associated nonneoplastic lymphoid stroma. A close relationship between EBV and LELC was reported in distinct clinicopathological entities such as EBV-associated gastric carcinoma, whereas inconsistent expression of EBV was observed in various organs [3]. Although LELC has been observed in many sites, primary LELC of the thyroid gland is extremely rare. The current case exhibited unusual cytomorphological patterns in FNAC and surgical specimens. The differential diagnoses included intrathyroid thymic carcinoma, metastatic LELC, medullary thyroid carcinoma, and anaplastic thyroid carcinoma (ATC) with LELC features. Stromal lymphocytic infiltration is one of the most characteristic features of intrathyroid thymic carcinoma, whose cells are polygonal epithelial cells with distinct nucleoli and an ill-defined cell border. Immunohistochemically, positive reactivity for CD5 is a key feature for differentiating intrathyroid thymic carcinoma from undifferentiated thyroid carcinoma [4]. Because medullary thyroid carcinoma has such a heterogeneous cell population, it should always be considered in differential diagnoses. Medullary carcinoma exhibits dispersed, pleomorphic cells (i.e., plasmacytoid, polygonal, and spindle cells), granular cytoplasm, and salt and pepper chromatin. Amyloid deposits are often observed in the background. However, LELC morphology with vesicular nuclei, distinct nucleoli, and extensively lymphocytic infiltration is inconsistent with the diagnosis of medullary carcinoma. Owing to the rare incidence of LELC in thyroid glands, this raises a concern regarding metastatic nasopharyngeal carcinoma. However, ATC often loses its expression of thyroid-specific markers such as thyroglobulin and TTF-1. Under such circumstances, excluding the metastatic origin can be difficult [5]. Clinical history and image findings have been reviewed and presented at a clinicopathological conference. According to clinical conclusions, metastatic disease cannot be verified. Therefore, primary thyroid carcinoma with LELC morphology has been proposed for this unusual malignancy.

Although the immunophenotype of our case indicated epithelioid–squamoid differentiation, the characteristic expression patterns of TTF-1, Pax-8, and CD5 were absent in the thyroid tumor. Notably, a remaining papillary carcinoma (PTC) component was observed in the contralateral metastatic nodes. The presence of concurrent PTC suggests a primary thyroid origin and a possible anaplastic transformation of PTC [5]. The left and right thyroid were extensively sampled for characteristic components of well-differentiated carcinoma. In this case, intrathyroid PTC was not discovered. Thus, the question remained whether metachronous thyroid cancer developed in different lobes. Based on a case series, metastatic nodal disease without identifiable thyroid primary is a rare but real phenomenon with unknown mechanisms [6]. Although most tumors are low grade and well differentiated, aggressive behavior due to poorly differentiated or anaplastic carcinoma can occur [6]. However, the current case exhibited a dense hyaline matrix within the LELC. Histopathologically, it has been speculated that such a matrix could represent “burnt out” residue from well-differentiated components. In the presenting patient, our assessment of LELC in association with PTC was the more commonly accepted diagnosis; however, we were unable to rule out the possibility of metachronous primary cancer in different thyroid lobes. Despite the unusual presentation, this thyroid tumor was distinctive because of the expression of EBV in EBER-ISH, which was indicative of EBV-related malignancy.

LELC has been designated as a subtype of ATC with epithelioid–squamoid morphology [5, 7]. ATCs are highly malignant tumors that histologically appear to wholly or partially comprise undifferentiated cells. The morphological spectrum depends on the admixture of three main histological patterns: spindle cell, giant cell, and squamoid cell [5, 7]. Locating well-differentiated or poorly differentiated thyroid carcinoma is possible in a considerable number of cases and is helpful for differential diagnosis. ATC constitutes less than 5% of clinically recognized thyroid malignancies but accounts for more than half of all deaths from thyroid cancer, with a mortality rate of over 90% and a mean survival of 6 months after diagnosis [5]. In a case review from the database of the Anaplastic Thyroid Carcinoma Research Consortium of Japan, researchers analyzed the histopathological features of ATC in patients who had achieved long-term survival [8]. The authors revealed histological findings distinct from the usual ATC features among the patients who had survived for more than 1 year. The presence of a pre-existing tumor, epithelial growth, squamous cell carcinoma component, increased lymphocytic infiltration, and lack of neutrophilic infiltration were the favorable prognostic factors in ATC [8]. Therefore, LELC morphology might play a role in clinical outcomes.

EBV is a well-established causative agent for a variety of malignant neoplasms. Its role in pathogenesis has been discussed in nasopharyngeal carcinoma, a subset of gastric carcinomas, EBV-associated smooth muscle tumors, and, to a lesser extent, inflammatory pseudotumor-like follicular dendritic cell tumors [3]. It was proposed that increased expression of EBV was related to dedifferentiation or progression from papillary to undifferentiated thyroid carcinoma [9]. EBV-associated thyroid carcinomas are seldom reported to have originated in the thyroid [9]. A favorable prognosis has been proposed in EBV-associated LELCs arising in the stomach, lung, liver, urinary bladder, salivary gland, and uterine cervix [10–15]. Regarding the thyroid, the behavior of EBV-related LELC has seldom been documented. As a low-grade thyroid carcinoma with a favorable outcome, intrathyroid thymic carcinoma is histologically similar to thymic carcinoma [4, 16]. The lack of evidence for the correlation between intrathyroid thymic carcinoma and EBV expression suggests that intrathyroid thymic carcinoma and LELC may be different in terms of etiology [17]. However, whether these rare thyroid malignancies belong to the same prognostic group requires further investigation.

## Conclusions

We reported a patient with an EBV-associated thyroid carcinoma exhibiting a characteristic LELC pattern. The patient had a positive response to radiotherapy with long-term survival in contrast to the lethal outcome of conventional anaplastic carcinoma. Because of its distinct histological features, association with EBV, and possible prognostic implication, awareness of this peculiar variant is vital for accurate clinicopathological diagnosis.

## Abbreviations

ATC: Anaplastic thyroid carcinoma
EBER-ISH: In situ hybridization for EBV encoded RNA
EBV: Epstein-Barr virus
FNAC: Fine-needle aspiration cytology
LELC: Lymphoepithelioma-like carcinoma
PTC: Papillary carcinoma
